# Retraction: MMP-13 is involved in oral cancer cell metastasis

**DOI:** 10.18632/oncotarget.10823

**Published:** 2016-07-25

**Authors:** Shun-Hong Huang, Ching-Hsuan Law, Ping-Hsueh Kuo, Ren-Yu Hu, Ching-Chieh Yang, Ting-Wen Chung, Ji-Min Li, Li-Hsun Lin, Yi-Chung Liu, En-Chi Liao, Yi-Ting Tsai, Yu-Shan Wei, Chi-Chen Lin, Chien-Wen Chang, Hsiu-Chuan Chou, Wen-Ching Wang, Margaret Dah-Tsyr Chang, Lu-Hai Wang, Hsing-Jien Kung, Hong-Lin Chan, Ping-Chiang Lyu

Authors and Oncotarget editors agree to retract the article “MMP-13 is Involved in Oral Cancer Cell Metastasis” after concerns regarding data duplication in multiple figures were confirmed through an internal investigation.

The authors of this manuscript extend their sincere apologies to the scientific community.

Original article: Oncotarget. 2016; 7(13): 17144-61. doi: 10.18632/oncotarget.7942.

